# Aphtha of the Gums

**Published:** 1879-10

**Authors:** 


### APHTHA OF THE GUMS.

TRANSLATED FROM THE FRENCH OF DR. DAVID.

The form of disease which we shall designate under that name is characterized by the development of aphtha upon the mucous membrane of the gums. The term aphtha, has not always had the same or precise signification which it has to-day. The ancient authors, (Livies, Hippocratique, Celse, etc.,) employed it for designating indifferently all of the ulcerations of the mucous membrane, and even gangrene of the mouth.

It is Van Swieten who first restricts the sense of it, in applying it only to the little ulcerations which come unexpectedly in the mouth, on a level with the mucous follicles.

In the compendium of medicine, is found the same confusion of ancient authors; because they apply the word aphtha, to all of the inflammatory affections of the mucous membrane of the mouth.

The classic contemporary authors, Grissolle, Tardieu, etc., agree, generally not to consider aphtha just as a simple, superficial ulceration, and limited to the mouth. Simonet, and after him, Hardy and Behier, make of it a tetter of the mucous membrane, (*un herpis des muquenses*).

In the meanwhile, Worms, who has made a more minute study of that simple affection returns to the idea of Van Swieten, and localises the affection at the orifice of the mucous glands, calling it *aeni des muquenses.* That which, according to that author, constitutes aphtha, is exuded from nature just as sebaceous matter, which arises from the mucous follicles.

As for us, we regard aphtha, with Hardy and Behier, as a simple superficial ulceration of the mucous membrane; and the term *d'herpis des muquenses*, expresses our idea exactly.

Indeed the explanation of Van Swieten, resumed by Worms, rests upon an anatomical error, that is to say, upon the supposed existence of glands in the mouth, or glands producing mucous. We have already insisted upon this point, *id. est.*, the mucous which covers the lips and the jaws, or cheeks, contains in lieu of a suit of glands, only isolated follicles, which secrete not a mucous, but a saliva, properly speaking. This saliva is different, according to the regions from which it is secreted, thus, viscid and thick in the bed of the mouth and upon the greater part of mucous membrane; and it is clear and limpid in the neighborhood of the mouth steno's duct, at the internal surface of the jaws.

As to the gum, properly speaking; it does not contain any gland, either mucous or saliva producing. Scarcely does one find there, towards the insertion of the labial fold, any glandules of the nature of those of the neighboring region. Aphtha ought then to be considered as a distinct lesion of the mucous membrane, without partaking whatever of the glands.

Very many of the classical authors have admitted in the eruption aphtheuse, the varieties *discrite et confluente*, (separate and running together). The first, is the one which we have solely in view in this article. The second has reference to the history of "*la stomatite generalisse.*" We shall find it, nevertheless, later, apropos of the gum of a pregnant woman, which takes the form of aphtha also sometimes. The seat of the aphtha of the gums varies according to different circumstances. In the child it is at the summit of the crest of the gum, which one recognises before the appearance of the first dentition; that was the opinion of Celse, repeated to-day by various authors. In the adult the outer surface of the gum seems to be reached exclusively. As for the remainder, that depends upon the etiological considerations into which we may enter. In the ordinary eruption aphtha, that which we see every day, appears to me to be an affection entirely local in its origin. And it is caused by the presence or introduction into the mouth of various irritant bodies. That which proves it, is, that aphtha shows itself precisely upon the part of the gum which is mostly exposed to the various irritants, such as tobacco, the pipe, aromatic aliments, in the case of the adult, upon the external surface of the gum.

In the infant it appears upon the crest of the gum, where the manœuvres of suction, during lactation, bear above all their action; and where it shows itself, the irritation coming solely from the eruption of the first teeth.

Things do not occur otherwise upon the mucous membrane of the mouth, where one recognizes only the influence of the direct irritants. Very recently we observed a young man who bore for very many years some aphtha upon the mucous membrane of the lower lip.

Being consulted one day with regard to this matter, we found opposite to the customary bed of the eruption, upon the necks of the teeth, antero-inferior, a large bed of tartar, which we did not hesitate to consider as the cause of the aphtha. The calcareous production was removed, and since then no eruption has been able to show itself upon the lip.

Aphtha, may be divided into two very distinct phases in its progress: The period of exudation under the ephithelium; and the period of ulceration.

The first is characterized by a small round vesicle, making relief very often appreciable. This vesicle, at first somewhat red and shining, becomes rapidly clear and transparent. In a very little while it swells up and bursts, allowing a colorless and watery liquid to issue forth. The pouch, just alluded to breaks the ephithelial partition, and then appears the small loss of substance, that is to say, the ulceration or better the erosion of the mucous membrane. If we wish to appreciate this loss of substance thoroughly, it is easy to convince ourselves by examining the border of the ulceration, which, in this instance, is not deep, and which interests only the epithelial bed. In wiping or cleansing it out well, one sees clearly the skin with all its papillary projections. One is able in that regard, to compare aphtha to a burn of the second degree, or to a process of blistering, in which, as every one knows the epidermis alone is found to be destroyed, and the dermis fully exposed to view. (*mis a un*).

Aphtha, the form of which is usually round, presents almost the dimensions of a lentil. (French lentille, which means a lance). It rarely goes beyond these dimensions, though this is far from the account given us of them of the ancient authors. It is in the last mentioned case that one may sometimes see the character of the syphilitic ulcers, from which it is sometimes very difficult to distinguish it. The main point of the ulcer is very often covered with a grayish coating which appears to be formed of epithelium, mixed with some globules of grease.

The eruption aphtha is ordinarily surrounded with a little red inflamed circle, which is not easily understood. The symptoms of aphtha are very much in accordance with anatomical lesions. The papillæ of the mucous membrane skin, exposed to view, are the seat of the living ulcer; above all when one practices the least touch with the fingers, with aliments, or may be even with the tongue, severe pain is experienced, and the mouth is used with difficulty.

These sufferings last until effectual relief is brought; that is to say, for four or five days on an average; when one does not modify the seat of the ulcer by any topical application. The loss of substance is filled up by the formation of a new epithelial bed, which takes all of the characteristic of the neighboring normal epithelium, and of a kind which the *aphtha* has left, absolutely, no trace. It is very curious to see the rapidity of that formation, in the track of a slight cauterization of the wound.

Everyone has seen the truth of this statement, that an *aphtha* touched in the evening by caustic, was, the following day, completely cicatrized, and did not occasion any other trouble or show any other sign.

*Aphtha* is a very superficial lesion, and always remains local. In habitual cases it has nothing corresponding to it in the lymphatic system which would serve one in making a diagnosis, for it is never accompanied by any general phenomena.

